# Long-Term Effects of Vegetative-Propagation-Mediated TuMV-ZR Transmission on Yield, Quality, and Stress Resistance in *Pseudostellaria heterophylla*

**DOI:** 10.3390/pathogens14040353

**Published:** 2025-04-05

**Authors:** Li Gu, Sheng Qian, Shuting Yao, Jiaxin Wu, Lianghong Wang, Jing Mu, Yankun Wang, Jianming Wang, Zhongyi Zhang, Mingjie Li

**Affiliations:** 1 College of Bee Science and Biomedicine, Fujian Agriculture and Forestry University, Fuzhou 350002, China; 2 Key Laboratory of Ministry of Education for Genetics, Breeding and Multiple Utilization of Crops, Fujian Agriculture and Forestry University, Fuzhou 350002, China; 3 National Resource Center for Chinese Materia Medica, State Key Laboratory for Quality Ensurance and Sustainable Use of Dao-di Herbs, Beijing 100700, China

**Keywords:** *P. heterophylla*, TuMV-ZR virus, vegetative propagation cycles, yield, quality, stress resistance

## Abstract

*Pseudostellaria heterophylla* (Miq.) Pax (*P. heterophylla*) was a valued traditional Chinese herbal medicine. Previous studies have shown that *P. heterophylla* TuMV spreads during the vegetative propagation cycle using tuberous roots as carriers. However, the transmission mechanism of TuMV in *P. heterophylla* and its effects on host growth remain to be elucidated. In this study, virus-free *P. heterophylla* culture seedlings were infected with control, TuMV-ZR, and TuMV-ZR-EGFP, thereby resulting in the initial infection cycle of IF1 (TIF1, TEIF1) and control NIF1, and used these roots to propagate the subsequent infection cycle IF2 (TIF2, TEIF2) and control NIF2. The transmission of TuMV-ZR seedlings was tracked by EGFP signal, and their yield, quality, and resistance were analyzed simultaneously in the critical growth period of the plants. The results indicated that TuMV-ZR accumulated in the tuberous roots of IF1 plants, subsequently migrated to IF2 during seedling growth, and was re-stored in IF2 tuberous roots, thereby forming a simple virus transmission cycle. Meanwhile, the tuberous roots of IF1 and IF2 *P. heterophylla* showed lower fresh weight, dry weight, soluble sugar, and saponin levels compared to NIF1 and NIF2, respectively. TuMV caused a significant reduction in chlorophyll synthesis in IF1 and IF2 *P. heterophylla*, resulting in impairment to their photosynthetic organs and efficiency. The measurement of stress resistance in IF1 and IF2 *P. heterophylla* revealed that continuous viral infection disrupted antioxidant enzyme activity, increased the content of MDA, enhanced the activity of PAL, and elevated the levels of intracellular osmotic substances in both propagation cycles. The findings indicated that the accumulation of the TuMV-ZR virus during two successive vegetative propagation cycles induced physiological stress, impaired photosynthesis, and caused progressive yield and quality decline with each cycle. This study systematically examined the impact of TuMV-ZR persistence during vegetative propagation on key physiological and biochemical indices in *P. heterophylla*, providing critical data to clarify vegetative-propagation-mediated germplasm degradation.

## 1. Introduction

*Pseudostellaria heterophylla* (*P. heterophylla*), a perennial herb belonging to the Caryophyllaceae family and the Pseudostellaria genus, has a significant medicinal value. This species is employed in the treatment of a wide range of health conditions including spleen deficiency, body fatigue, anorexia, lung dryness, and dry cough. Consequently, it stands out as a vital raw material in traditional Chinese medicine [1]. With the rising demand for *P. heterophylla*, its cultivation has expanded significantly in several Chinese provinces, including Fujian, Guizhou, Jiangsu, and Anhui. However, viral pathogens are prevalent, with disease incidence ranging from 60% to 90%, sometimes up to 100% [2,3]. Furthermore, the primary propagation method used in the production of *P. heterophylla* involves vegetative propagation using tuberous roots. This method facilitates the direct transmission of viruses to subsequent vegetative propagation cycles, significantly contributing to the widespread persistence of viral diseases. As a result, the incidence of viral diseases in *P. heterophylla* has been increasing annually, posing a significant threat to the yield and quality of this plant and leading to the degradation of high-quality *P. heterophylla* varieties [4,5]. Consequently, there is an urgent need to identify the causative pathogens and implement effective control measures.

Recent advances have revealed slight regional variations in virus species composition across various production areas. An analysis of virus types and their distribution ratios indicates that the primary pathogens responsible for the disease are TuMV (turnip mosaic virus), BBWV2 (broad bean wilt virus 2), and CMV (cucumber mosaic virus). Among these, TuMV has been identified as the most prevalent virus associated with the disease [2,3,6]. The species TuMV from *genus Potyvirus,* family Potyviridae, constitutes one of the most abundant groups of plant RNA viruses, representing approximately 30% of all known plant viruses [7]. TuMV demonstrates an extensive host range, infecting approximately 320 plant species across 156 genera in 43 families, including cruciferous, labiate, compositae, leguminosae, and caryophyllaceae crops [7]. Consequently, this virus is regarded as one of the most significant challenges to the safe-mannered production of agricultural crops [8,9]. In a previous study, the complete genome sequence of TuMV in *P. heterophylla* was identified and designated as TuMV-ZR [6]. Based on this, TuMV-ZR and TuMV-ZR-EGFP infectious clones were constructed and optimized for *P. heterophylla* [6]. These clones provide a valuable tool for tracking single-virus transmission and studying the physiological and biochemical effects on host plants.

Viral disease transmission, as demonstrated in the field of animal epidemiology, is principally categorized into vertical and horizontal transmission. In vegetatively propagated plants, viral particles are not transmitted by the classical horizontal or vertical transmission routes but are passively propagated along with host tissues during tuber or tuberous root division [10,11]. Unlike sexually propagated plants where seed-based regeneration can purge viruses, clonal propagation perpetuates viral loads across cycles. Vegetative-propagation-mediated virus maintenance thus leads to progressive viral accumulation in vegetative organs, exacerbating more severe and lasting damage over time to result in germplasm degradation, characterized by irreversible declines in genetic stability, agronomic traits, and stress resilience [12,13]. In *P. heterophylla*, sustained TuMV-ZR maintenance across tuberous root-propagation cycles directly correlates with severe yield loss and quality deterioration. The specific mechanisms of viral transmission across successive generations and their damaging effects on *P. heterophylla* remain to be elucidated, significantly hindering the development of effective viral control strategies; thus, analyzing these mechanisms and their impacts is essential for preventing viral diseases and mitigating germplasm degradation.

In this study, the TuMV-ZR virus, identified as the core pathogen of the *P. heterophylla* virus disease, was utilized as the subject. TuMV-ZR and TuMV-ZR-EGFP infectious clones were employed to infect virus-free *P. heterophylla* seedlings, generating initial infection plants containing this virus. The harvested tuberous roots were used as propagation material to grow subsequent propagation cycles, ensuring that each vegetative propagation cycle contained the respective viruses in their tuberous roots. This process established populations of infected plants over two successive vegetative propagation cycles. On this basis, key indicators, including yield and quality, photosynthetic characteristics, antioxidant enzyme activity, osmotic adjustment substances, and stress-related enzyme activity, were analyzed in two vegetative propagation cycles of *P. heterophylla* at a crucial virus infection stage. By evaluating these indicators, this analysis aimed to assess the virus’s impact on the physiological and biochemical functions of *P. heterophylla*. The findings enhance our understanding of virus-induced germplasm degradation and support the development of strategies to prevent and control viral diseases.

## 2. Materials and Methods

### 2.1. Planting and Management of Experimental Material

**Figure 1:**
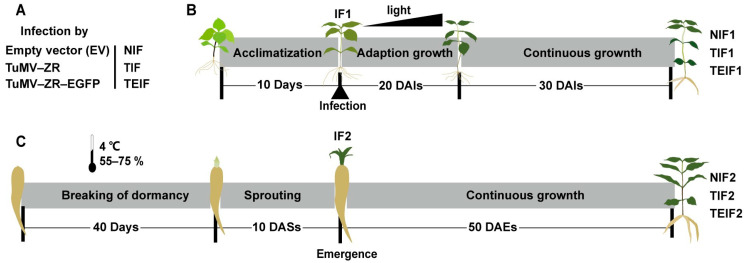
Schematic representation of TuMV-ZR infection cycles in *P. heterophylla*. Virus-free tissue-cultured seedlings (10-day acclimated) served as initial infection material. These were inoculated with wild-type TuMV-ZR, recombinant TuMV-ZR-EGFP, and empty vector control, establishing first-generation infected (IF1: TIF1/TEIF1) and non-infected (NIF1) *P. heterophylla* (**A**). At 50 days after inoculation (DAIs), IF1 and NIF1 plants developed tuberous roots (**B**). Following dormancy termination, these roots were transplanted into soils, initiating bud elongation (**C**). By 10 days after sprouting (DASs), emerged shoots established second-generation infected (IF2: TIF2/TEIF2) and non-infected (NIF2) populations (**C**). Subsequent tuberous root formation at 50 days after emergence (DAEs) completed the infection cycle (**C**).

Virus-free tissue-cultured seedlings from “Zheshen No. 1” variety bred by the Zherong County Agricultural Technology Extension Service Center in Fujian Province, China, along with *Nicotiana benthamiana* (*N. benthamiana*), were used as experimental materials to assess viral systemic infection patterns. These materials were cultivated in a free virus isolation greenhouse at the GAP Research Institute of Fujian Agriculture and Forestry University. The propagation of infectious clone pCB301 (empty vector), pCB301-TuMV-ZR, and pCB301-TuMV-ZR-EGFP was achieved using *N. benthamiana* for the preparation of *P. heterophylla* TuMV-ZR virus particles. The *N. benthamiana* was cultivated in a nutrient soil-based substrate under controlled conditions: a 12 h/12 h photoperiod (day/night), a temperature of 25 °C, and a light intensity of 6000-7000 Lux. In a subsequent experiment, virus-free tissue-cultured seedlings of *P. heterophylla*, ranging in age from 30 to 45 days, were acclimated to a photoperiod of 12 h/12 h (day/night) with a light intensity of 500 Lux. This was carried out to allow full adaptation to the cultured environment (Figure 1).

Three infectious clone preparations derived from *N. benthamiana* were utilized to infect *P. heterophylla* seedlings, thereby establishing the initial infection groups (IF1). The IF1 group comprised two treatments: TuMV-ZR infected (TIF1) and TuMV-ZR-EGFP infected *P. heterophylla* (TEIF1). The control initial non-infected *P. heterophylla* (NIF1) was employed as a comparison to assess the effects of viral infections. These IF1 and NIF1 seedlings were then exposed to gradually increasing light intensities, ranging from 500 to 6000 Lux, with the final intensity being reached at 6000 Lux (Figure 1).

The expansion of tuberous roots in IF1 and NIF1 *P. heterophylla* seedlings produced the initial cycle of infected tuberous roots (TF1r and TEF1r) and non-infected tuberous roots (NIF1r). Following the breaking of dormancy by means of low-temperature treatment, the IF1r and NIF1r were transferred to a nutrient soil-based substrate under a photoperiod of 12 h/12 h (day/night) with light intensities of 6000 Lux. This led to the subsequent propagation cycle of infected *P. heterophylla* (TIF2 and TEIF2) and non-infected *P. heterophylla* (NIF2), as well as the formation of their corresponding tuberous roots (NIF2r, TIF2r, and TEIF2r) (Figure 1). The infectious clones of TuMV-ZR and TuMV-ZR-EGFP utilized in this study were maintained at a temperature of −80 °C.

### 2.2. Preparation of Virus Particles of P. heterophylla TuMV-ZR Virus

The infection of *N. benthamiana* and *P. heterophylla* was conducted in accordance with the virus infection method for *P. heterophylla*, as previously established by Yang et al. (2023) [6]. Specifically, the *Agrobacterium tumefaciens* (*A. tumefaciens*) strains with TuMV-ZR and TuMV-ZR plasimids were streaked onto resistance plates containing kanamycin and rifampicin. Positive *A. tumefaciens* clones were selected from above resistance plates and cultivated in 50 mL of liquid LB medium under 180 rpm with 28 °C. Once the OD600 value of the *A. tumefaciens* solution reached 1.0, the bacteria were harvested by centrifugation at 4000× *g* for 15 min, after which the bacterial pellets were re-suspended in a mixture of 10 mM MES (pH 5.6), 10 mM MgCl_2_, and 200 µM acetosyringone. Seedlings displaying five to six leaves were selected for infection, and the infection solution was loaded into a 1 mL needle-free syringe for injection into the abaxial side of the second and third leaves of *N. benthamiana*.

### 2.3. Inoculation of P. heterophylla TuMV-ZR Virus Particles in P. heterophylla

After confirming the full spread of the two TuMV-ZR viruses in *N. benthamiana* plants via RT-PCR, a total of 100 mg of virus-infected leaves was carefully excised and ground into a homogenate using 2 mL of virus extraction buffer and 20 mg of carborundum [6]. For the infection experiment, the third and fourth leaves of virus-free *P. heterophylla* culture seedlings with five to six leaves were selected. Cotton swabs dipped in the grinding solution were used to gently rub the abaxial surfaces of these leaves. Following a 24-h dark treatment, the plants were transferred to a virus isolation infection room for further cultivation. The infection process in *P. heterophylla* was monitored by tracking EGFP-tagged TuMV-ZR fluorescence using a handheld fluorescence excitation lamp with 440–460 nm excitation and 500 nm emission wavelength. To minimize background interference, fluorescence signals from non-infected control roots were captured under identical settings.

### 2.4. Validation of Virus Levels and Infection States in Virus-Infected P. heterophylla

Total RNA from different treatments was isolated using TRIZOL methods. In brief, 100 mg of tissue samples were subjected to grinding in liquid nitrogen, followed by homogenization with TRIZOL solution. Following the phase separation of chloroform and the precipitation of the RNA using isopropanol, the RNA was purified through a series of ethanol washes. The purified RNA pellet was then dissolved in DEPC-treated water for concentration and purity assessment. The presence of viral infection in different treatments of *P. heterophylla* was confirmed using the RT-PCR method [6], identifying positive plants with corresponding virus. Total RNA was extracted from the leaves and roots of three *P. heterophylla* treatments after root enlargement, and reverse transcription was performed using the HiScript II 1st Strand cDNA Synthesis Kit (Vazyme Biotech Co., Ltd., Nanjing, China). Highly conserved sequence primers CP-Infec-Detec-F and CP-Infec-Detec-R [6], targeting the TuMV-ZR coat protein (CP) gene, were used to detect the virus infection status in both leaves and roots of *P. heterophylla*. Furthermore, the previously developed TuMV-ZR TaqMan qPCR method [3] was applied to quantify the virus levels in the leaves and roots following different treatments, providing precise viral quantification.

### 2.5. Breaking Dormancy of Tuberous Roots of Virus-Infected P. heterophylla

The dormancy of *P. heterophylla* tuberous roots was broken by low-temperature treatment in order to accelerate sprouting; thereafter the stems, leaves and fibrous roots were detached from the tuberous roots. Briefly, the residual tuberous roots were then carefully cleaned using sterilized water, followed by a thorough drying process, in preparation for subsequent processing. The roots were then placed in a sterilized cultivation substrate (nutrient soil: vermiculite = 4:1) and enclosed in self-sealing bags, which were then sealed tightly. These bags were then refrigerated at a temperature of 4 °C for 40 to 50 days, during which time the relative humidity was maintained at a range of 55–75%. During this period, the humidity levels and the sprouting status of terminal buds were closely monitored. The process of accelerating sprouting was considered to be complete once the elongation of terminal buds reached approximately 1 cm. The roots bearing developed buds were then extracted from the low-temperature environments.

### 2.6. Morphological Observation and Biomass Measurement of Virus-Infected P. heterophylla

The phenotypic characteristics of *P. heterophylla* subjected to various treatments were systematically documented through photographic records. Additionally, the EGFP fluorescence in TEIF1 and TEIF2 *P. heterophylla* was examined under using a handheld fluorescence excitation lamp (440–460 nm excitation, 500 nm emission filter). During the critical growth and development periods, several key indices were recorded. The number of leaves and roots for each treatment was meticulously recorded. Plant height was measured using a standard scale. A vernier caliper was employed to accurately measure the diameter of the roots in different treatments. An analytical balance was used to determine the fresh weight of the aboveground portions and the fresh and dry weights of the tuberous roots for each treatment.

### 2.7. Determination of Physiological Indices of Virus Treated P. heterophylla

During the critical growth and development period of *P. heterophylla*, root and leaf samples were collected from *P. heterophylla* treated with TuMV-ZR. Each treatment was biologically replicated three times and immediately frozen in liquid nitrogen before being stored at −80 °C. Approximately 2 g of root or leaf tissues were homogenized in 3 mL of 50 mM potassium phosphate buffer (pH = 7.0). The resultant homogenate was centrifuged at 10,000× *g* for 10 min at 4 °C to obtain the filtrate, which was used for determining various physiological indicators.

The NBT (5,5-dietyl-4-amino-2,5-dione), the guaiacol, and the ultraviolet absorption method were utilized to assess the activity of superoxide dismutase (SOD), as well as peroxidase (POD) and catalase (CAT), respectively [14]. The thiobarbituric acid (TBA), the ninhydrin colorimetry, and the anthrone colorimetry methods were used to determine malondialdehyde (MDA) and proline soluble and sugar contents [15,16]. Finally, the phenylalanine ammonia lyase (PAL) assay was conducted according to the Herui Bioassay Kit protocol (Herui Biotechnology Co., Ltd., Fuzhou, China). The determination of chlorophyll concentration in leaves was accomplished by employing the ethanol–acetone extraction method [17]. The quantification of total polysaccharide and total saponin contents in the roots of *P. heterophylla* was achieved through the utilization of the phenol–sulfuric acid and the vanillin–perchloric acid chromogenic method, respectively [18,19]. A series of chlorophyll fluorescence parameters were measured using a modulated chlorophyll fluorescence imaging system (M-IMAGING-PAM, Walz GmbH, Effeltrich, Germany). These parameters included Fv/Fm (maximum quantum yield of PSII photochemistry), Y (II) (effective quantum yield of PSII), ETR (electron transport rate), NPQ (non-photochemical quenching), and qN (quenching of variable fluorescence).

### 2.8. Data Statistics and Analysis

GraphPad Prism 9.3.0 software (San Diego, CA, USA) was employed to visualize and plot various biomass and physiological indicators from different treatments. The statistical analysis of the measured physiological index data was conducted using DPS 7.05 software (Hangzhou Ruifeng Information Technology Co., Ltd., Hangzhou, China). All data are expressed here as mean ± SD of three independent biological replicates. Statistical significance was determined by one-way ANOVA followed by Fisher’s LSD test (*p* < 0.05).

## 3. Results

### 3.1. Tracing of TuMV-ZR Virus Transmission in Initial Virus Infection Cycle of P. heterophylla

**Figure 2:**
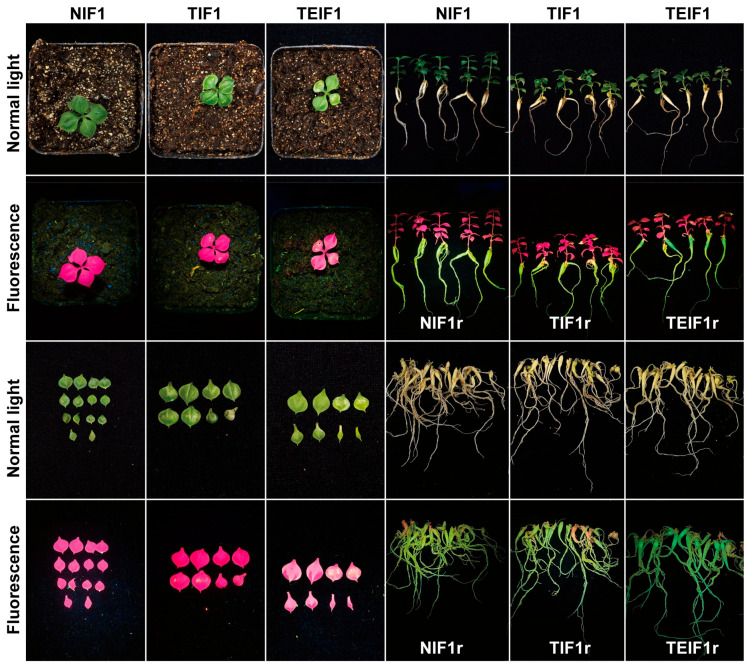
Phenotypic symptoms of the roots, leaves, and whole plants of IF1 (TIF1 and TEIF1) and NIF1 *P. heterophylla* during the critical infection stages of TuMV-ZR virus (50 days after inoculation, DAIs). This figure illustrates the visible effects of viral infection on different organs of the plants, highlighting changes in morphology and development states under normal and blue light excitation.

The TuMV-ZR virus reaches peak infection in *N. benthamiana* at 20 days after inoculation (DAIs), with leaves from this stage being used for virus preparation. To investigate the transmission of the TuMV-ZR virus during *P. heterophylla* vegetative propagation, three treatments (control, TuMV-ZR, and TuMV-ZR-EGFP) were employed to generate the initial cycle of virus-infected *P. heterophylla* (IF1: TIF1 and TEIF1) and control non-infected *P. heterophylla* (NIF1) by infecting virus-free tissue culture seedlings. Subsequent propagation cycles of infected (IF2: TIF2 and TEIF2) and non-infected *P. heterophylla* (IF2) were obtained using tuberous roots from IF1 *P. heterophylla*. An extensive viral spread was observed in *P. heterophylla* plants at 35–50 DAIs. The clear green fluorescence in TEIF1 was noted while NIF1 and TIF1 did not exhibit similar emissions. This finding was basically consistent with the results of previous studies [6]. Notably, by 50 DAIs, a significant accumulation of clear green fluorescence was observed in the tuberous roots of TEIF1 *P. heterophylla* (Figure 2). *P. heterophylla* plants at this stage were thus selected for the study of the TuMV-ZR infection status, its quantity, and the effects on the growth of *P. heterophylla*.

Viral presence in NIF1, TIF1, and TEIF1 at 50 DAIs was confirmed by RT-PCR using TuMV-specific primers targeting the viral CP gene. The results obtained indicated that TIF1 and TEIF1 leaves and roots were amplified to the expected product length of approximately 500 bp whereas NIF1 samples did not amplify corresponding products (Figure S1). This finding thus confirmed the presence of TuMV-ZR in TIF1 and TEIF1. The subsequent quantification of viral expression in the leaves and roots of *P. heterophylla* was conducted utilizing a TaqMan RT-qPCR assay. The results confirmed no detectable viral presence in NIF1 leaves and roots whereas TIF1 leaves and roots showed substantial viral loads of 4.69 × 10^6^ copies/μL and 2.90 × 10^5^ copies/μL, respectively (Table S1). The TEIF1 samples exhibited 6.42 × 10^5^ copies/μL and 4.26 × 10^5^ copies/μL, respectively (Table S1).

### 3.2. The Transmission of TuMV-ZR Virus in Subsequent Infection Cycle of P. heterophylla and Its Effect on Phenotypic Characteristics of Plants

**Figure 3:**
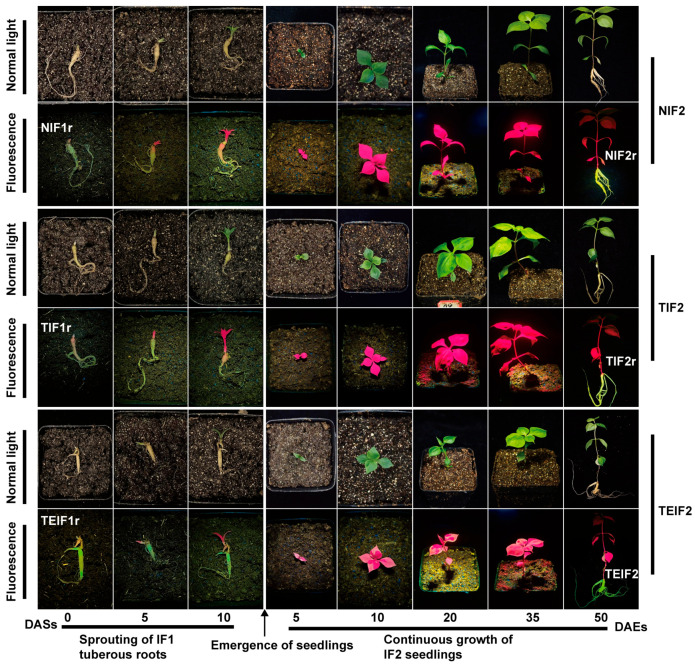
Sprouting process of tuberous roots from IF1 *P. heterophylla* and phenotypic characteristics of IF1 *P. heterophylla* during growth. The apical buds of the tuberous roots from IF1 *P. heterophylla* began to sprout after its dormancy was broken. These buds emerged above the soil surface, forming the IF2 *P. heterophylla*. Following further development, these IF2 *P. heterophylla* plants eventually developed into new tuberous roots with TuMV-ZR. This figure shows the changes in appearance characteristics from IF1r to IF2r.

Following the breaking of dormancy in the tuberous roots of NIF1, TIF1, and TEIF1 (designated as NIF1r, TIF1r, and TEIF1r), the next generation of infected (NIF2, TIF2) and virus-free *P. heterophylla* (TEIF2) was propagated vegetatively from tuberous root cuttings. After sprouting from the tuberous roots, green fluorescence was observed in TEIF1r roots whereas no fluorescence was detected in TIF1r and NIF1r (Figure 3). By 5 days after sprouting (DASs), significant bud elongation was evident across all treatments. By 10 DASs, *P. heterophylla* seedlings across all three treatment groups emerged with visible shoot elongation, marking the developmental transition from the IF1 to the IF2 stages (Figure 3). At 10 days after emergence (DAEs), the viral infection in the tuberous roots of IF2 *P. heterophylla* began to migrate towards the leaves. By 20 DAEs, shoots of the TEIF2 demonstrated accelerated growth, accompanied by the rapid propagation of green fluorescence in TEIF2 leaves. Between 35 and 50 DAEs, the green fluorescence had extensively spread throughout the entire TEIF2 plants (Figure 3).

At 50 DAEs, NIF2, TIF2, and TEIF2 were removed from the cultivation substrates for further analysis. It was observed that the IF2 tuberous roots of each treatment expanded in size. Notably, in TEIF2, green fluorescence was detected within the expanded roots, indicating potential virus accumulation in the tuberous roots (TEIF2r) (Figure 3). In contrast, the tuberous roots of NIF2 and TIF2 (TIF2r and NIF2r) showed no fluorescence (Figure 3). Furthermore, the presence of green fluorescence-tagged TuMV-ZR was distinctly evident during the storage and dormancy phases of TEIF2r (Figure S2). RT-PCR confirmed the presence of virus in the leaves and tuberous roots of TIF2 and TEIF2 but not in NIF2, as evidenced by the amplification of PCR products matching the theoretical length (Figure S3). This confirmed the presence of the virus in the IF2 *P. heterophylla*, indicating virus accumulation in the new cycle’s tuberous roots (IF2r). Concurrently, the expression levels of the virus in the leaves and roots from the three treatments were determined using TaqMan qPCR. The resulting TuMV-ZR levels in NIF2 leaves and roots were found to be close to zero whereas TuMV-ZR levels in TIF2 leaves and roots reached 1.24 × 10^8^ copies/μL and 8.31 × 10^5^ copies/μL, respectively (Table S1). TuMV-ZR levels in TEIF2 leaves and roots reached 9.20 × 10^7^ copies/μL and 8.31 × 10^5^ copies/μL, respectively (Table S1). This comprehensive analysis demonstrates the progression of viral infection through vegetative propagation and its effects on the growth and development of IF2 *P. heterophylla*, confirming that the virus accumulated in the roots of TEIF2 and TIF2 but not in NIF2.

### 3.3. The Effects of TuMV-ZR Infection Across Successive Vegetative Propagation Cycles on the Yield and Quality of of P. heterophylla

**Figure 4:**
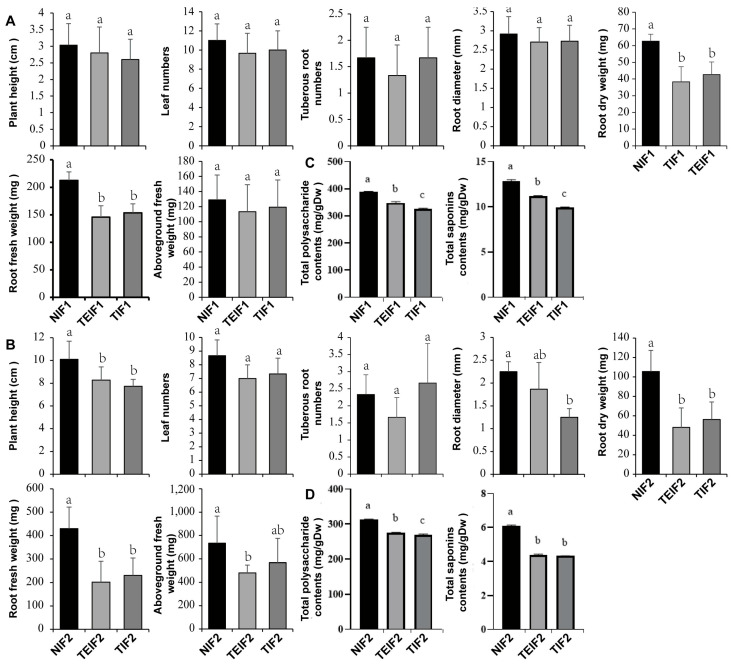
Differences in biomass (**A**,**B**) and quality indicators (**C**,**D**) between IF1 (TIF1 and TEIF1) and NIF1 *P. heterophylla* at 50 days post-inoculation (DAIs) following TuMV-ZR virus infection and IF2 (TIF2 and TEIF2) and NIF2 *P. heterophylla* after 50 days after emergence (DAEs). Data are represented as mean ± SD (n = 3 plants per group). Lowercase letters denote statistically significant differences between groups (*p* < 0.05, LSD test).

To investigate the effect of sustained virus infection on the growth of *P. heterophylla*, some growth and biomass indices were measured for NIF1, TIF1, and TEIF1 and NIF2, TIF2, and TEIF2 *P. heterophylla*. In IF1 *P. heterophylla*, both TIF1 and TEIF1 infections resulted in a significant decline in biomass compared to NIF1 infection. Specifically, TIF1 showed reductions of 27.7% and 31.9% in fresh weight and dry weight, respectively (Figure 4A). A similar trend was observed in TEIF1, with reductions of 31.5% and 38.8% in corresponding indices, respectively, compared to NIF1 (Figure 4A). The effects of viral infection were subsequently evaluated in IF2 *P. heterophylla*. The results demonstrated that TIF2 exhibited significant reductions in root fresh weight, root dry weight, and aboveground fresh weight by 46.4%, 46.8%, and 22.6%, respectively, in comparison to NIF2 (Figure 4B); TEIF2 exhibited a substantial decline, with reductions of 53.1%, 54.3%, and 34.3% respectively in the same indices (Figure 4B). In addition, both TIF2 and TEIF2 exhibited significantly diminished plant heights and root diameters in comparison to NIF2 (Figure 4B). These results indicate that there was a significant suppression of biomass in both IF1 and IF2 *P. heterophylla* due to a persistent TuMV-ZR infection. The detrimental effects of persistent viral infection on *P. heterophylla* were highlighted by significant reductions in growth parameters and biomass.

At the same time, the effect of persistent viral infection on the active components (polysaccharides and saponins) in the tuberous roots of *P. heterophylla* was investigated. The results showed that the total contents of polysaccharides and saponins in the tuberous roots of TIF1 and TEIF1 were significantly lower than those in NIF1 (Figure 4C). In particular, marked reductions in polysaccharide and saponin contents were observed in TIF1 when compared to NIF1, with respective decreases of 15.9% and 22.9% (Figure 4C). A similar trend was observed for TEIF1 in comparison to NIF1, in which the two active components exhibited reductions of 10.3% and 13.4%, respectively (Figure 4C). The subsequent analysis focused on the determination of the total amounts of polysaccharides and saponins in the tuberous roots of IF2. The results showed that TIF2 had a 14.1% lower polysaccharide content and 29.1% lower saponin content compared to NIF2 (Figure 4D). Similarly, TEIF2 showed a reduction of 12.2% in the polysaccharide content and a reduction of 28.0% in the saponin content compared to NIF2 (Figure 4D).

### 3.4. Effects of TuMV Transmission Across Successive Vegetative Propagation Cycles on the Photosynthetic Characteristics of P. heterophylla

**Figure 5:**
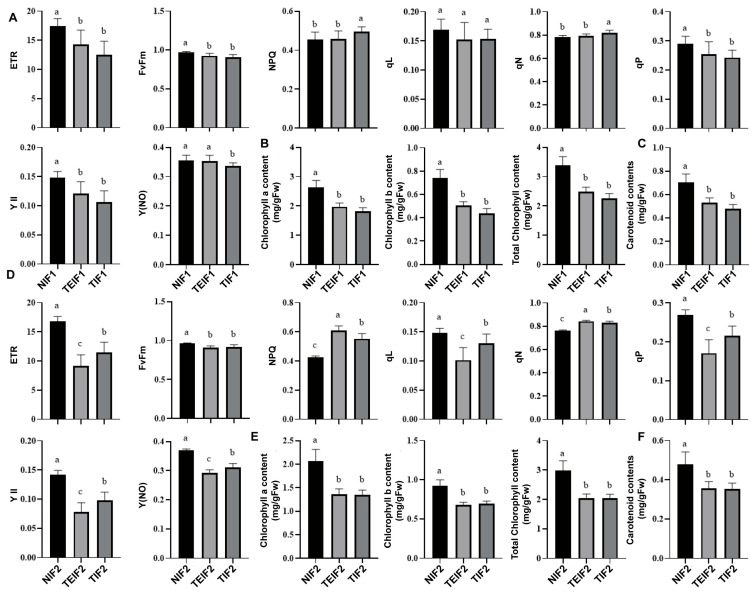
Effects of TuMV-ZR virus infection on chlorophyll fluorescence parameters (**A**,**D**), as well as on chlorophyll a, chlorophyll b, total chlorophyll levels (**B**,**E**), and carotenoid levels (**C**,**F**) in IF1 (TIF1 and TEIF1), NIF1, IF2 (TIF2 and TEIF2), and NIF2 *P. heterophylla*. Data are represented as mean ± SD (n = 3 plants per group). Lowercase letters denote statistically significant differences between groups (*p* < 0.05, LSD test).

To analyze the effect of TuMV-ZR infection on the photosynthetic efficiency of *P. heterophylla*, the chlorophyll fluorescence parameters of *P. heterophylla* were measured after virus infection. In comparison to NIF1, the parameters Fv/Fm, YII, ETR, Y(NO), qP, and qL showed a significant decrease in TIF1 and TEIF1 leaves whereas NPQ and qN showed a significant increase (Figure 5A). Simultaneously, the chlorophyll-related indicators were measured in NIF1, TIF1, and TEIF1 leaves. The results showed that chlorophyll a, chlorophyll b, total chlorophyll, and carotenoid contents in TIF1 were significantly lower than in NIF1 by 31.2%, 41.1%, 33.3% and 21.9%, respectively (Figure 5B,C). Similarly, the corresponding indices for TEIF1 were reduced by 25.2%, 32.0%, 26.7%, and 24.9%, respectively, compared to those in NIF1 (Figure 5B,C). Further measurements of chlorophyll fluorescence parameters in NIF2, TIF2, and TEIF2 leaves showed that Fv/Fm, YII, ETR, Y(NO), qP, and qL were also significantly reduced in TIF 2 and TEIF 2 leaves compared with NIF 2 whereas NPQ and qN were significantly elevated (Figure 5D). Further analysis focused on the chlorophyll a, chlorophyll b, total chlorophyll, and carotenoid contents in IF2. In addition, the results showed that the four corresponding chlorophyll indices of TIF2 were significantly lower than those of NIF2 by 35.0%, 24.7%, 31.8%, and 26.6%, respectively (Figure 5E,F). The corresponding four indices of TEIF2 were also lower than those of NIF2 by 34.5%, 26.4%, 32.0%, and 25.8%, respectively (Figure 5E,F). These results indicate that continuous TuMV-ZR infection not only reduces photosynthetic pigment levels but also affects intergenerational photosynthetic performance.

### 3.5. Effects of TuMV-ZR Infection on the Antioxidant Enzyme of Initial and Subsequent Infection Generation of P. heterophylla

**Figure 6:**
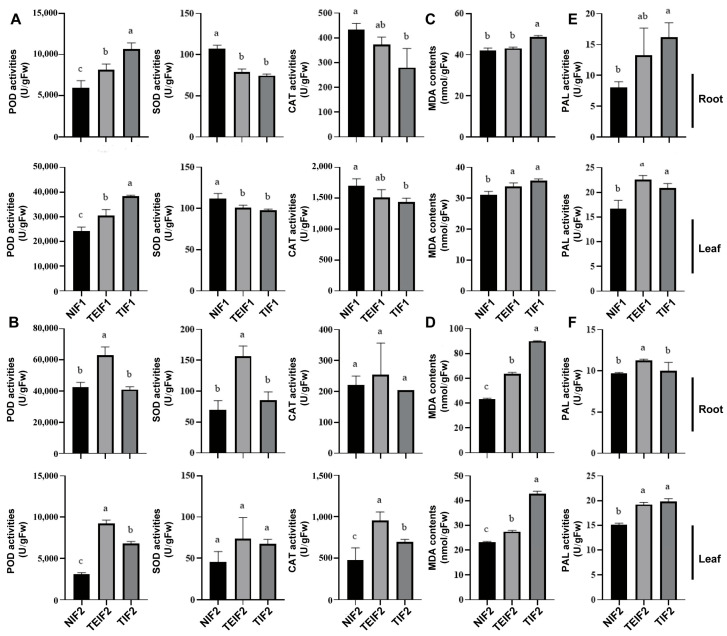
Effects of TuMV-ZR virus infection on antioxidant enzyme activities (**A**,**B**), MDA content (**C**,**D**), and PAL activity (**E**,**F**) in the roots and leaves of IF1 (TIF1 and TEIF1), NIF1, IF2 (TIF2 and TEIF2), and NIF2 *P. heterophylla*. Data are represented as mean ± SD (n = 3 plants per group). Lowercase letters denote statistically significant differences between groups (*p* < 0.05, LSD test).

To investigate the effect of TuMV-ZR infection on the antioxidant enzyme activity of *P. heterophylla*, the antioxidant enzyme activities were determined in NIF1, TIF1, and TEIF1 plants. The results showed that superoxide dismutase (SOD) and catalase (CAT) activities were significantly decreased in both the leaves and roots of TIF1 and TEIF1 compared to NIF1 whereas peroxidase (POD) activity was significantly increased (Figure 6A). The subsequent analysis focused on the determination of the activities of SOD, POD, and CAT in the roots and leaves of NIF2, TIF2, and TEIF2, respectively. In comparison with NIF2, the activities of SOD, CAT, and POD were significantly increased in the roots of TIF2 and TEIF2. In the leaves, the activities of SOD and POD in TEIF2 showed a significant increase whereas the activity of CAT remained largely unchanged (Figure 6B). However, the changes in three antioxidant activities in TEIF2 were not significantly different from those in NIF2. The results indicate that, while TIF1 and TEIF1 *P. heterophylla* demonstrated reductions in SOD and CAT activities, alongside increased POD activity in response to TuMV-ZR infection, TIF2 and TEIF2 *P. heterophylla* presented a more complex pattern of antioxidant enzyme responses.

### 3.6. Effects of TuMV-ZR Infection Across Successive Vegetative Propagation Cycles on MDA Content and PAL Activity of P. heterophylla

The levels of malondialdehyde (MDA) and the activity of the polyphenol oxidase (PAL) enzyme were measured in the leaves and roots of NIF1, TIF1, and TEIF1. The results demonstrated that, in comparison to NIF1, TIF1 and TEIF1 infection resulted in a significant increase in MDA levels in both leaves and roots. Among them, TIF1 and TEIF1 infection significantly increased MDA contents in leaves of *P. heterophylla* (Figure 6C); in roots, MDA contents were significantly increased only in TIF1. The subsequent determination of MDA contents and PAL activity in the leaves and roots of NIF2, TIF2, and TEIF2 showed that the MDA contents in the roots and leaves of TIF2 and TEIF2 was significantly higher than that in NIF2 (Figure 6D). At the same time, the PAL activity of roots and leaves showed significant changes in TIF1 and TEIF1 (Figure 6E). Among them, the PAL contents in the leaves of TIF1 and TEIF1 plants increased significantly compared to the control, and only the PAL activity in the roots of TIF1 plants increased significantly. PAL activity in TIF2 and TEIF2 was significantly higher than that in NIF2 in leaves, but in roots, only TIF2 PAL activity was significantly higher than control (Figure 6F). The results indicate that virus infection significantly increased MDA levels and PAL activity in TIF1, TEIF1, TIF2, and TEIF2, suggesting that the virus continued to exert a significant stress effect on both two-infection cycles of *P. heterophylla*.

### 3.7. Effect of TuMZ-ZR Infection Across Successive Vegetative Propagation Cycles on the Content of the Osmotic Substances of P. heterophylla

**Figure 7:**
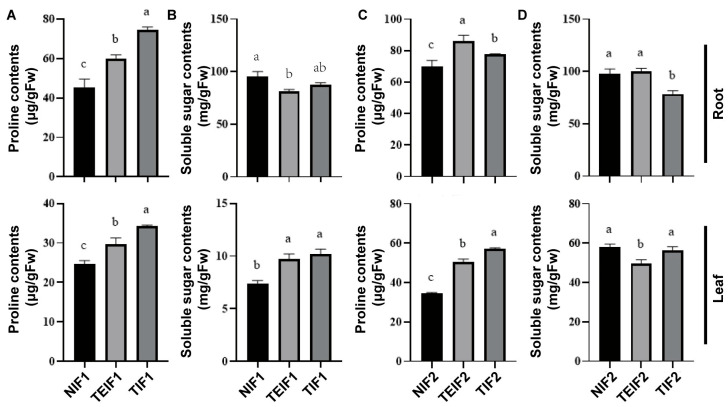
Effects of TuMV-ZR virus infection on proline contents (**A**,**C**) and soluble sugar contents (**B**,**D**) in the roots and leaves of IF1 (TIF1 and TEIF1), NIF1, IF2 (TIF2 and TEIF2), and NIF2 *P. heterophylla*. Data are represented as mean ± SD (n = 3 plants per group). Lowercase letters denote statistically significant differences between groups (*p* < 0.05, LSD test).

To gain a more profound understanding of the impact of virus infection on the osmotic substances in *P. heterophylla*, an investigation was conducted into the changes in soluble sugar and proline concentrations in the leaves and roots of IF1 and IF2 *P. heterophylla*. The results of the investigation demonstrated that the levels of proline and soluble sugars in the leaves of TIF1 and TEIF1 were significantly higher than those in NIF1 (Figure 7A,B), thereby providing a clear indication of the differences in osmotic substance levels for two viruses. Furthermore, the investigation of proline and soluble sugar contents in the roots revealed a significant increase in TIF1 and TEIF1 in comparison to NIF1 whilst the soluble sugar content was found to be significantly lower (Figure 7A,B). With respect to proline, the proline levels in the roots and leaves of TIF2 and TEIF2 were found to be significantly higher than those in NIF2 (Figure 7C). In addition, the effects on IF2 *P. heterophylla* were investigated, and the results showed that soluble sugars in the roots of TIF2 decreased and the leaves of TEIF1, in comparison to NIF2 (Figure 7D). In summary, these findings indicate that virus infection not only alters the concentration and distribution of two osmotic substances in both two vegetative propagation cycles of *P. heterophylla*. This suggests a significant stress response to viral infection that impacts both cycles’ physiological states.

## 4. Discussion

For the vegetative propagation of plants, the transmission of viruses through vegetative organs has a significant impact on the growth and development of host plants across successive vegetative propagation cycles. Extensive studies have demonstrated that there is a substantial increase in the incidence of viruses in the field with successive vegetative cycles, which further results in discriminating degradation and significant reductions in yield and quality. The continuous spread of viral diseases has thus emerged as a critical limiting factor in the successful cultivation of vegetative propagation crops. *P. heterophylla*, a typical vegetative propagation plant, has also been found to be significantly impacted by the continuous spread of viral diseases in the field [20]. Previous studies have shown that the *P. heterophylla* virus can accumulates in significant quantities in the tuberous roots of the host, thereby identifying tuberous roots as a key element contributing to the transmission of the virus and the exacerbation of viral diseases [3,6]. Therefore, understanding viral accumulation in *P. heterophylla* tuberous roots and analyzing the physiological responses mediated by the viruses are crucial for elucidating the damage and germplasm degradation mechanisms caused by continuous virus transmission.

Building on previous studies [3,6], this study mapped the transmission pathways and patterns of TuMV-ZR-EGFP during the vegetative propagation of *P. heterophylla*. It specifically focused on elucidating the transmission dynamics of TuMV-EGFP between the initial and subsequent vegetative propagation generations, with tuberous roots serving as the medium for virus transmission. qPCR assays revealed that the viral level of TuMV-ZR-EGFP was consistently lower than that of the wild-type TuMV-ZR, likely due to reduced replication efficiency caused by the insertion of the EGFP marker gene. This finding provided direct evidence that prolonged vegetative propagation drives viral accumulation in tuberous roots, a process central to virus-mediated germplasm degradation. However, it is crucial to recognize that viral disease-induced germplasm degradation in *P. heterophylla* production often requires several years to become evident. This study detected no significant germplasm degradation spanning only two successive vegetative propagation cycles. Further qPCR analysis demonstrated progressively elevated viral titers of TuMV-ZR in tuberous roots across cycles, indicating that increasing cycles of vegetative propagation may accelerate viral accumulation, potentially leading to phenotypic degradation. To confirm this hypothesis, future studies should involve repeatedly propagating infected tuberous roots across multiple vegetative propagation cycles while simultaneously quantifying virus levels and examining the relationship between virus accumulation and germplasm degradation.

Virus infection has a significant adverse impact on yield, quality, and growth in plants [21,22]. The analysis of *P. heterophylla* in initial (IF1) and subsequent vegetative propagation cycles (IF2) has shown that TuMV-ZR infection reduces dry weight, the fresh weight of tuberous roots, and the contents of polysaccharides and saponins. These findings indicate that virus transmission disrupts biomass accumulation and key metabolite production. Virus diseases manifest with leaf symptoms like distortion, discoloration, and mosaic patterns [23], reducing photosynthetic efficiency and overall plant productivity. Studies show that viral infections decrease chlorophyll content and photosynthetic rates [24,25,26]. This study demonstrated that TuMV infection caused a substantial decrease in chlorophyll (a and b) and total chlorophyll contents, along with carotenoid levels, in *P. heterophylla*. This decline would inevitably result in impaired photosynthesis and inhibited metabolite accumulation. Chlorophyll fluorescence parameters critically assess photosynthetic efficiency, where Fv/Fm reflects PSII’s maximum light–energy conversion efficiency, Y(NO) quantifies photodamage risk, qP/qL indicates PSII reaction center openness/connectivity, and Y(II)/ETR measures actual photosynthetic activity [27,28]. These indices collectively evaluate photosynthetic performance, photoprotective mechanisms, and stress adaptation dynamics. TuMV-ZR infection in *P. heterophylla* (IF1/IF2) significantly elevated NPQ/qN while suppressing Fv/Fm, Y(II), ETR, qP, qL, and Y(NO) in both IF1 and IF2 *P. heterophylla*. This suggests that viral infection inhibits PSII photochemical reactions and electron transfer, damages photosynthetic structures, and reduces photosynthetic rates. The findings provide a comprehensive understanding of the impact of TuMV infection on the photosynthetic process in both IF1 and IF2 *P. heterophylla*, aligning with established virus–host interaction mechanisms [29,30,31]. Consequently, an analysis of yield and photosynthetic characteristics in two cycles of *P. heterophylla* post viral infection indicates that continuous virus transmission during vegetative propagation disrupts photosynthesis, leading to sustained reductions in yield and quality.

Viral diseases propagate intracellularly via nuclear entry and systemic tissue invasion, inflicting cumulative cellular damage. Unlike acute pathogens causing rapid mortality, viruses establish chronic infections that chronically dysregulate plant physiology [32,33]. Deciphering these protracted virus–plant interactions is therefore critical for elucidating viral pathogenesis mechanisms. Key antioxidant enzymes (SOD, POD, CAT) constitute frontline defenses against biotic stress, with activity modulations quantitatively reflecting infection severity [34]. Studies in various plants have shown that viral infections significantly affect antioxidant enzyme activities [29,35,36]. This study revealed that viral transmission damages the antioxidant system of *P. heterophylla*, with IF1 *P. heterophylla* showing increased POD and decreased CAT and SOD levels, similar to other virus-infected plants. However, these enzymes show different changes in IF2 *P. heterophylla*, likely due to varying levels of virus infection and the difference of plant responses. In IF1 *P. heterophylla*, a shorter initial infection time leads to stronger stress responses. In IF2 *P. heterophylla*, prolonged viral presence results in the antioxidant enzyme adaptation of *P. heterophylla* to the virus. In addition, virus-free seedlings in IF1 might own relatively lower resistance compared to tuberous root seedlings in IF2. MDA, a by-product of ROS-induced membrane damage from antioxidant enzyme disorder, is a reliable indicator of plant damage [37]. Virus infections significantly increase MDA levels, causing serious membrane damage [38]. Although the antioxidant enzyme system displayed inconsistent alterations between IF1 and IF2 *P. heterophylla*, the substantial increases in MDA levels across both vegetative propagation cycles were indicative of a disrupted oxidation equilibrium, accompanied by notable cell membrane impairment.

In processes of virus infections against various plants, the disruption of viruses to cell membranes accelerates the decomposition of cellular substances, leading to an accumulation of small molecules such as soluble sugars and proline [39]. These compounds have been shown to help regulate osmotic pressure. The presence of soluble sugars and proline in response to virus infection has been widely documented [40,41]. These substances are regarded as vital indicators of stress response in plants, and the analysis of their concentrations allows for the precise estimation of virus-induced damage. In this study, a substantial increase in soluble sugars and proline was observed in *P. heterophylla*, suggesting that continuous viral spread in both two-infection cycles of *P. heterophylla* had resulted in severe damage to *P. heterophylla*. In the interaction between plants and pathogens, PAL metabolism is a pivotal secondary pathway. This pathway is activated during biotic stress, leading to the production of defensive substances such as phenols, phenyl derivatives, lignin, and flavonoids [42]. These substances combat pathogens by means of this pathway, which plays a pivotal role in pathogen defense and serves as a key indicator of stress response. TuMV-ZR infection has been observed to significantly increase PAL activity in both the roots and leaves of *P. heterophylla*, suggesting that viral infection alters PAL metabolism, promoting the synthesis of downstream metabolites and lignin and affecting cellular metabolic activity. Consequently, TuMV-ZR infection has been shown to notably impact the life activities of both vegetative propagation cycles of *P. heterophylla*, triggering various defense responses to mitigate stress.

## 5. Conclusions

This study found that the virus is transmissible between successive vegetative propagation cycles of *P. heterophylla* through tuberous roots, with a significant impact on yield and quality during the vegetative propagation cycles of TuMV-ZR infected *P. heterophylla*. Continuous viral spread disrupted the photosynthetic system, reduced photosynthetic efficiency, and hindered the accumulation of photosynthetic products. In addition, TuMV-ZR infection significantly altered antioxidant enzyme and PAL activities in both virus-infected cycles, leading to excessive MDA accumulation, membrane lipid damage, induction, and increases in osmotic substances and other physiological stress. These damages and stresses were evidenced to compromise the overall vitality and productivity of the plant, resulting in corresponding decreases in quality and yield. Moreover, the ongoing damage was not only persistent but also demonstrated increasing trends as the virus disseminated. This study offered a reference point for understanding the mechanisms of virus-induced plant damage and provided physiological insights into the process of the virus-mediated degradation of the *P. heterophylla* germplasm.

## Data Availability

The original contributions presented in this study are included in the article/Supplementary Materials. Further inquiries can be directed to the corresponding author.

## Supplementary Materials

The following supporting information can be downloaded at: https://www.mdpi.com/article/10.3390/pathogens14040353/s1, Table S1: Analysis of TuMV-ZR virus levels in leaves and roots of both initial and subsequent virus infection generation of *P. heterophylla* infected with TuMV-ZR, using the TaqMan qPCR method; Figure S1: Detection of virus infection states in NIF1, TEIF1, and TIF1 *P. heterophylla* by RT-PCR method; Figure S2: Detection of EGFP-tagged TuMV-ZR in tuberous roots during storage and dormancy breaking of TEIF1 *P. heterophylla*. Figure S3: Detection of virus infection states in NIF2, TEIF2, and TIF2 *P. heterophylla* by RT-PCR method.
