# Supplementary material for: Long-Term Effects of Vegetative-Propagation-Mediated TuMV-ZR Transmission on Yield, Quality, and Stress Resistance in Pseudostellaria heterophylla
Source: Pathogens. 2025 Apr 5;14(4):353. doi: 10.3390/pathogens14040353 (PMC12030326; doi:10.3390/pathogens14040353)
Supplement: Supplementary file 1 [file pathogens-14-00353-s001.zip › pathogens-3484211-supplementary.pdf]

Table S1. Analysis of TuMV-ZR virus levels in leaves and roots of both initial and subsequent virus infection generation of *P. heterophylla* infected with TuMV-ZR, using the TaqMan qPCR method.

Organisms	Virus Infection Treatment	Biological Replicate 1	Biological Replicate 2	Biological Replicate 3	Mean	Standard Deviation (SD)
**IF1**						
	NIF1	NA	NA	NA	NA	NA
Leaf	TEIF1	610,997	1,151,457	163,276	641,910	494,815.25
	TIF1	11,783,965	1,980,320	309,720	4,691,335	6,198,933.5
	NIF1	NA	NA	NA	NA	NA
Root	TEIF1	130,755	1,136,510	11,211	426,158.67	618,079.25
	TIF1	728,859	109,611	31,680	290,050	382,012.19
**IF2**						
	NIF1	NA	NA	NA	NA	NA
Leaf	TEIF1	138,329,998	74,854,763	62,750,184	91,978,315	40,595,432
	TIF1	288,784,842	51,807,237	30,188,981	123,593,687	143,467,507
	NIF1	NA	NA	NA	NA	NA
Root	TEIF1	1,080,989.5	257,384.81	1,155,763.6	831,379.3	498,497.8
	TIF1	2,237,157.5	180,479.67	76,526.03	831,387.74	1,218,541.4

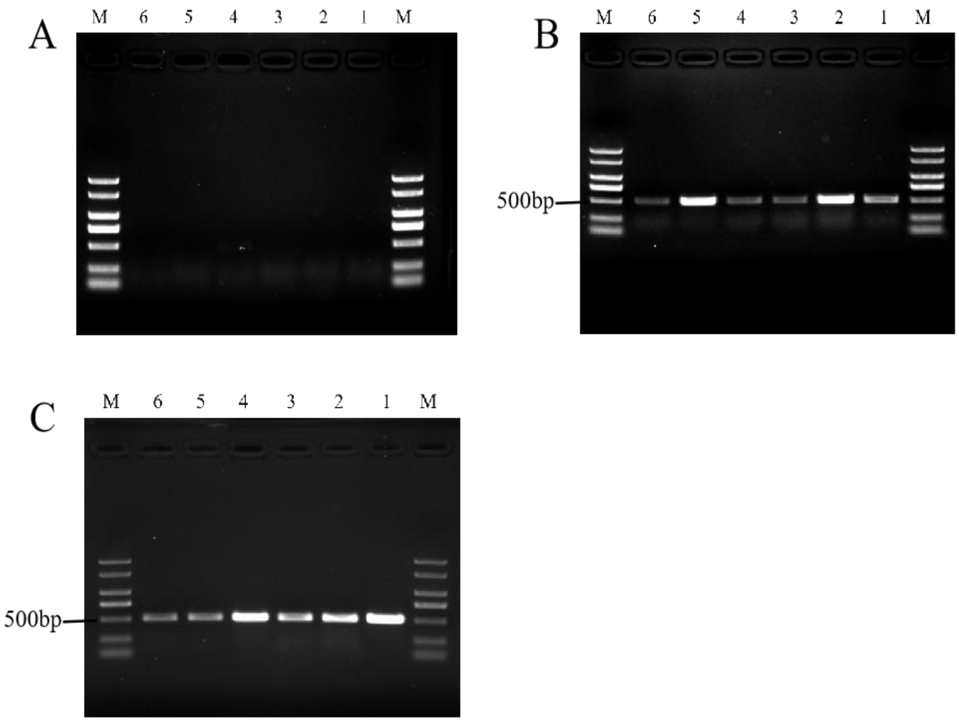

Figure S1. Detection of virus infection states in NIF1 (A), TEIF1 (B), and TIF1 (C) *P. heterophylla* by RT-PCR method. In the figure, Lane M represents DNA Maker 2000, while Lane 1, 2 and 3 represent the leaves of *P. heterophylla*, and Lane 4, 5 and 6 represent the roots of *P. heterophylla*.

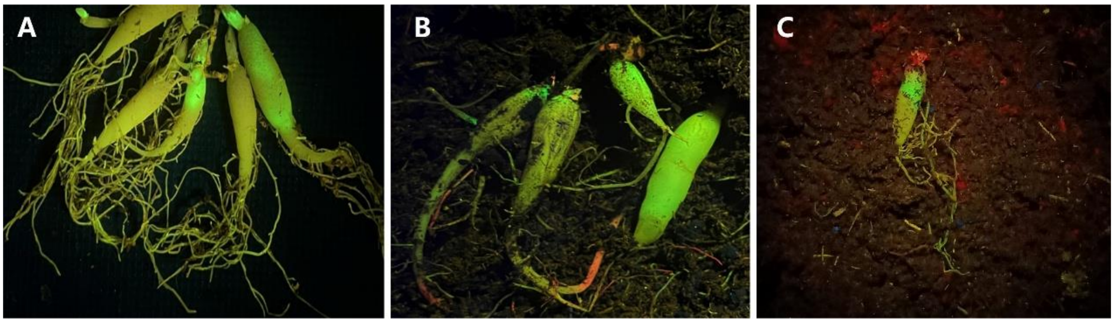

Figure S2. Detection of EGFP-tagged TuMV-ZR in tuberous roots during storage and dormancy breaking of TEIF1 *P. heterophylla*. Scattered fluorescence from TuMV-ZR-EGFP is clearly visible in harvested (A), stored and dormant (B), and sprouted (C) samples from TEIF1 *P. heterophylla*, indicating the presence and activity of the virus in these samples.

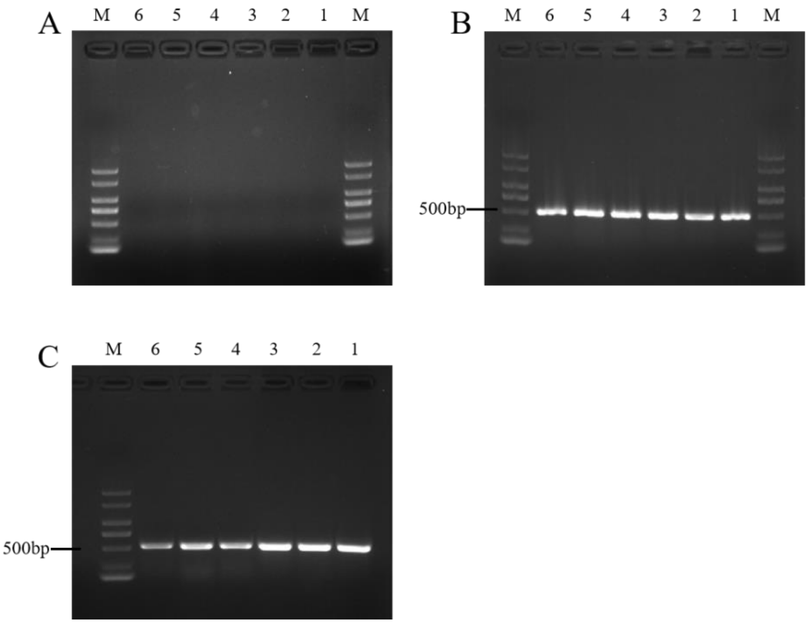

Figure S3. Detection of virus infection states in NIF2 (A), TEIF2 (B), and TIF12(C) *P. heterophylla* by RT-PCR method. In the figure, Lane M represents DNA Maker 2000, while Lane 1, 2 and 3 represent the leaves of *P. heterophylla*, and Lane 4, 5 and 6 represent the roots of *P. heterophylla*.
